# Supplementary material for: Identification of photoperiod-induced specific miRNAs in the adrenal glands of Sunite sheep (Ovis aries)
Source: Front Vet Sci. 2022 Jul 22;9:888207. doi: 10.3389/fvets.2022.888207 (PMC9354845; doi:10.3389/fvets.2022.888207)
Supplement: Supplementary file 1 [file Data_Sheet_1.ZIP › Supplementary file/miRDeep2 file.zip!/miRDeep2 file/Know_Structure/LP42A1/oar-mir-410.pdf]

miRBase precursor : oar-mir-410
Total read count : 639
oar-miR-410-5p read count : 2
oar-miR-410-3p read count : 634
remaining reads : 3

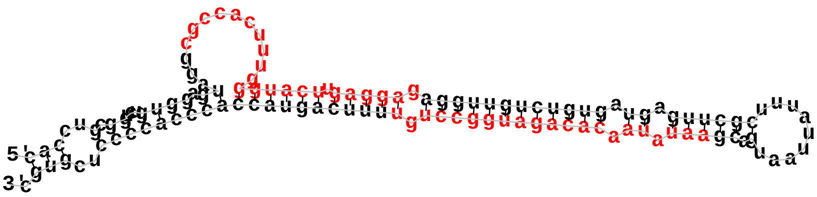

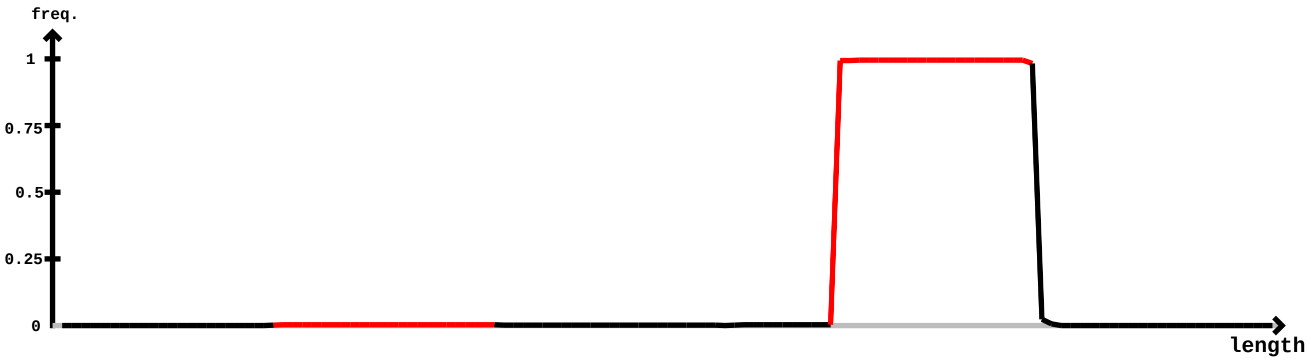

oar-miR-410-5p

oar-miR-410-3p

```
5'- caccugcggucuguggaguaggcgccacuuuggguacuugaggagagguugucugugaugaguucgcuuuauuaaugacgaauauaacacagauggccuguuuucaguaccacccaccccucgugc   -3'    exp
    (((..(.(((..((((........))))....((((((.(((((.((((((((((((.((.((((((.........).))))).)).))))))))))))).)))))))))))....))).)..))).    reads    mm
    .....................gcgccacuuuggguacuugaggag.................................................................................    1        0
    ......................cgccacuuuggguacuugaggag.................................................................................    1        0
    .............................................agguugucugugaugaguucgcu..........................................................    1        0
    .....................................................................uauuaaugacgaauauaacacagauggccugu.........................    1        0
    ......................................................................auuaaugacgaauauaacacagauggccuguuu.......................    1        0
    ................................................................................aauauaacacagauggccug..........................    8        0
    ................................................................................aauauaacacagauggccugu.........................    612      0
    ................................................................................aauauaacacagauggccuguu........................    11       0
    ................................................................................aauauaacacagauggccuguuu.......................    2        0
    ..................................................................................uauaacacagauggccuguuu.......................    1        0
```
